# Proposed New Club for Nurses

**Published:** 1919-03-22

**Authors:** 


### Proposed New Club for Nurses.

The Lord Mayor of London (Sir Horace B. Marshall, M.A.) presided on Thursday last at a Mansion House meeting held to promote a scheme to found a club in connection with the Royal British Nurses' Association.

In opening the proceedings, the Lord Mayor said it seemed to him that this period of armistice was not only a fitting opportunity to inaugurate such a scheme as that before them, but to impress upon the City of London the desirability of paying a tribute of regard for the conspicuous services which nurses have rendered to the nation. There was a debt of gratitude due to nurses, and the scheme showed how that debt could be paid, so far, at any rate, as concerned those connected with the Royal British Nurses' Association. Before the war, said a great surgeon, the position of nurses was a hard one, and the recognition they had since received was inadequate to the value of the aid they rendered. Hard as surgeons and medical men had worked during the war, nurses had worked harder. In commending this scheme he did not forget that there is a club in existence in connection with the College of Nursing which was doing a work of great value, but there was a need also for the particular club now brought before them. The nurses did not ask for charity. When the building was forthcoming the Club would be self-supported by the nurses themselves. He moved that the meeting cordially approved the scheme and would do all it could to further its success.

Mrs. Lloyd George, seconding, said she was sure the public needed no stimulation where nurses were concerned. Thousands of men are alive to-day simply because British nurses at a critical time stuck to their posts. Their work would bear comparison with that of either soldiers or sailors during the war, and while others were receiving public recognition nurses should not be overlooked.

Lord Morris, speaking in support, said there was not a soldier or sailor whom misfortune had landed in a hospital who did not come out with words of praise for the "ministering angels" there. The work of the nurse to-day was almost as important as that of the physician or the surgeon, and the more special and technical became the work of the medical man, so must the skill and technique of the nurse increase. For that reason alone, apart from national gratitude, the public should support this scheme. Let them put up a magnificent building that would be a monument for all time. This was a great opportunity for some of the wealthy of the land to put their hands deep into their pockets.

The Marchioness of Ailsa described how £10,000 had been raised in Glasgow to found a Nurses' Club there, and referred to the "unadvertised devotion" of nurses, which, so far, had met with little reward.

Dr. McGregor Robertson spoke of nurses as "feckless bodies" where their own interests were concerned. They showed unwillingness to exert themselves in their own behalf, and therefore the public must do it for them. Competent, healthy, and contented nurses were a national asset.

Admiral Sims, U.S.N., said he happened to have two sisters who were trained in that great institution, the Johns Hopkins Hospital in Baltimore, and he remembered how distressed he was when they told him that the nurses had to work twelve hours at a stretch—that the day was divided into a sort of two watches of twelve hours each. That was not right or wise. People who ran hospitals should come to sailormen for advice on that point. They did not ask a man to remain on the bridge of a great ship more than four hours at a time; to do so would be to run risk of disaster.

The motion was carried with acclamation.

